# Proteomic profiling of non-ischemic myocardium following acute mechanical cardiac unloading in ischemic heart failure

**DOI:** 10.1016/j.jmccpl.2026.100878

**Published:** 2026-10-29

**Authors:** Spyros A. Mavropoulos, Changwon Kho, Marika Fava, Ahyoung Lee, Kyra Leonard, Samta Veera, Tomoki Sakata, Renata Mazurek, Anjali J. Ravichandran, Jonas M. Marx, Xiaoke Yin, Manuel Mayr, Kiyotake Ishikawa

**Affiliations:** a Cardiovascular Research Institute, Icahn School of Medicine at Mount Sinai, New York, NY, USA; b Division of Applied Medicine, School of Korean Medicine, Pusan National University, Yangsan, Republic of Korea; c National Heart and Lung Institute, Imperial College London, London, United Kingdom; d Research Institute of Korean Medicine, Pusan National University, Yangsan, Republic of Korea

**Keywords:** Myocardial infarction, Left ventricular unloading, Proteomics

## Abstract

**Background:**

The cardiac proteome responds to various physiological and pathological stimuli. While acute left ventricle (LV) unloading by catheter-based LV assist device is now a therapeutic option for heart failure, its impact on cardiac proteins is unknown. We sought to characterize proteomic remodeling associated with altered LV load in ischemic heart failure.

**Methods:**

Sixteen Yorkshire pigs underwent myocardial infarction (MI) and were subjected one week later to either 2-hour acute mechanical LV unloading (*n* = 6), 2-hour LV overloading with mechanically-induced acute aortic regurgitation (*n* = 4), or no load interventions (*n* = 6). Non-ischemic remote myocardium was processed for proteomics. Age-matched naïve pigs (*n* = 6) served as reference. Protein abundance was analyzed using four-group LIMMA moderated ANOVA and pairwise LIMMA moderated *t*-tests. These were followed by functional enrichment and STRING functional association network analysis of ANOVA-significant proteins together with exploratory screening for load-associated candidates.

**Results:**

A total of 1713 proteins were identified, of which 1552 were included in the statistical analysis, and 7 remained significant after multiple-testing correction. Pairwise analyses revealed that significant protein-level differences were predominantly observed between the MI and Sham groups, whereas protein changes in response to 2-h of acutely altered LV load were limited. An exploratory screening identified six load-associated candidate proteins, with ITIH1 and MYOZ2 selected as representative candidates. Independent human transcriptomic analysis suggested disease-related context of these candidates.

**Conclusions:**

Proteomic remodeling of the non-ischemic remote myocardium in response to acutely altered LV load was less prominent compared to the impact of MI. Exploratory analysis identified LV load-associated candidate proteins.

## Highlights

• Proteins in non-ischemic remote myocardium actively respond to MI and altered cardiac load in a clinically relevant model of HF.

## Graphical abstract

Ischemic heart failure pig models were used to investigate how alterations in cardiac loading conditions affect protein expression in the non-ischemic remote myocardium. Proteomic analysis revealed less significant changes in proteins after 2 h of acutely altered load compared to the changes before and after MI. Exploratory analysis identified two LV load-associated protein candidates for further investigation.

**Figure:**
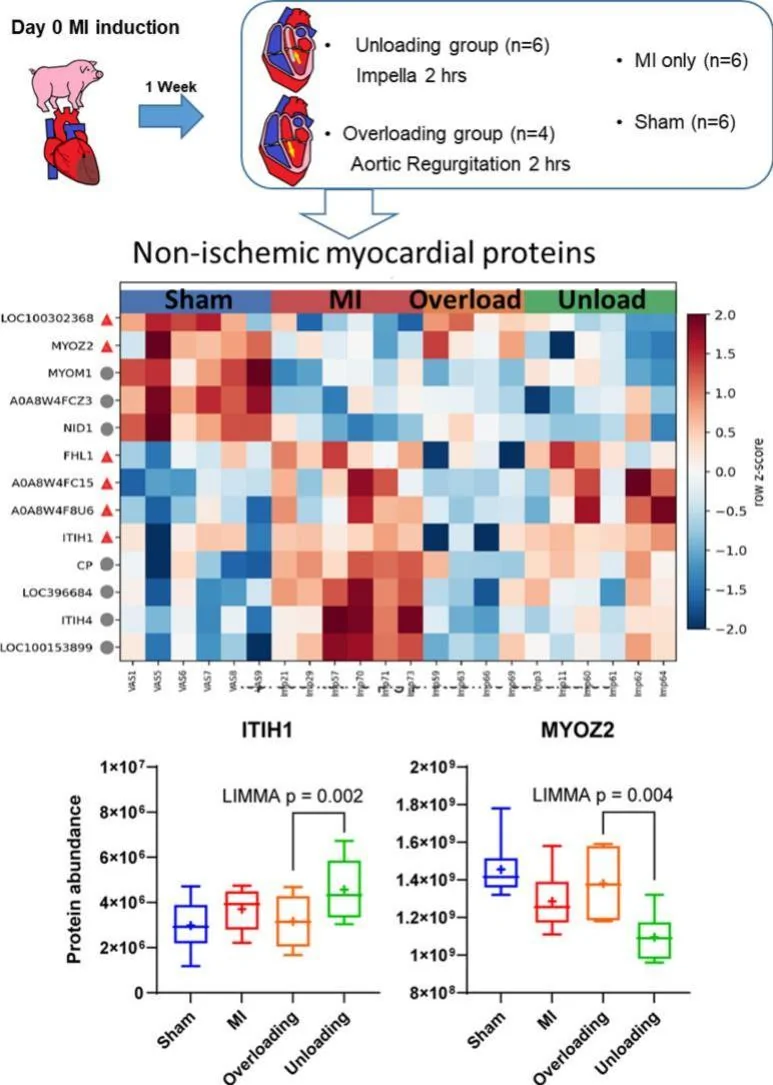

## 1 Introduction

Mechanical left ventricular (LV) unloading using surgical LV assist devices (LVAD) induces reverse cardiac remodeling and functional improvement in a subset of patients with severe LV dysfunction [1]. Previous studies have investigated the mechanisms underlying this myocardial recovery [2], [3], [4], [5] and have identified several potential contributors including restoration of calcium-handling proteins [6], improved mitochondrial function [7], and extracellular matrix remodeling [8]. More recently, advances in mechanical circulatory support technology have enabled short-term catheter-based approaches of LV unloading, referred to as acute mechanical LV unloading. Percutaneous LVAD provides hemodynamic improvement and relieves elevated wall stress by decreasing LV volume and pressure [9], [10]. It also reduces myocardial oxygen consumption and corrects heart failure-induced derangements in cardiac metabolism [11], [12]. Recent clinical study demonstrated improved survival with percutaneous LVAD support in cardiogenic shock patients [13]. However, despite the increasing clinical use of acute mechanical LV unloading, associated myocardial changes remain poorly characterized, largely due to difficulty in obtaining human myocardial tissues during short-term mechanical support. A better understanding of the myocardial adaptations induced by acute mechanical LV unloading may provide new opportunities to further optimize this therapeutic strategy. We hypothesized that LV myocardial protein expression is actively regulated in response to changes in cardiac loading conditions and that these protein changes are associated with beneficial effects of acute LV unloading. To examine this hypothesis, we used a swine model of subacute myocardial infarction (MI) and conducted proteomic analysis of the non-ischemic remote LV myocardium after acute alterations in LV loading. To further characterize the impact of acute mechanical LV unloading, we also included animals undergoing acute LV overloading as the opposite intervention, thereby enabling a gain-of-function and loss-of-function comparison.

## 2 Methods

### 2.1 Animal models of LV unloading and overloading

All animal procedures were approved by the Institutional Animal and Use Committee at the Icahn School of Medicine at Mount Sinai, and the care of animals complied with the Guide for the Care and Use of Laboratory Animals (National Institutes of Health publication No. 85-23, revised, 1996). The animals were acclimatized to the facility for at least 72 h before being enrolled in experiments.

A schematic of the experimental design is shown in Fig. 1. For proteomic study, twenty-two Yorkshire pigs (*Sus scrofa*) (8 males, 14 females; 40.0 ± 7.3 kg) were pre-medicated using intramuscular Telazol (tiletamine/zolazepam) (8.0 mg/kg), intubated and ventilated with 100% oxygen. General anesthesia was maintained with intravenous propofol (10 mg/kg/h) throughout the procedure. Buprenorphine (0.03 mg/kg) was given prior to the procedure for analgesia. Ten pigs were subjected to 90-min balloon occlusion of the middle left anterior descending artery and subsequent reperfusion and recovery, as previously described [14]. These animals were randomized to load alteration studies 1 week later. For this proteomic study, a third group (MI; *n* = 6, 37.1 ± 8.8 kg) of age-matched pigs that only underwent MI induction were enrolled and served as a reference, while a fourth group (Sham; *n* = 6, 35.7 ± 7.9 kg) that did not undergo either MI or unloading/overloading was used as a normal heart reference set. One week after MI induction, ten pigs for load alteration groups underwent baseline functional measurements with three-dimensional echocardiogram and LV pressure catheter. Subsequently, they were randomly assigned to either the Unloading group (*n* = 6) or Overloading group (*n* = 4). For the Unloading group, mechanical LV support was initiated using an Impella CP (Abiomed, Danvers, Massachusetts). Fluoroscopy was used to ensure proper positioning of the device, and intravenous heparin was given for anticoagulation. Pump support was increased stepwise, and maximal achievable support was maintained in each pig for 2 h. For the Overloading group, moderate to severe aortic regurgitation was induced by mechanical disruption of the aortic valve using myocardial biopsy forceps. Animals were euthanized at the end of protocol under unloaded or overloaded state by intravenous injection of 20 mEq potassium solution under deep isoflurane anesthesia. The heart was explanted immediately, immersed in cold cardioplegic solution, and tissue pieces collected frozen for further analysis. Infarct scar size was evaluated by staining the remaining slices with 1% Triphenyltetrazolium chloride.

**Fig. 1:**
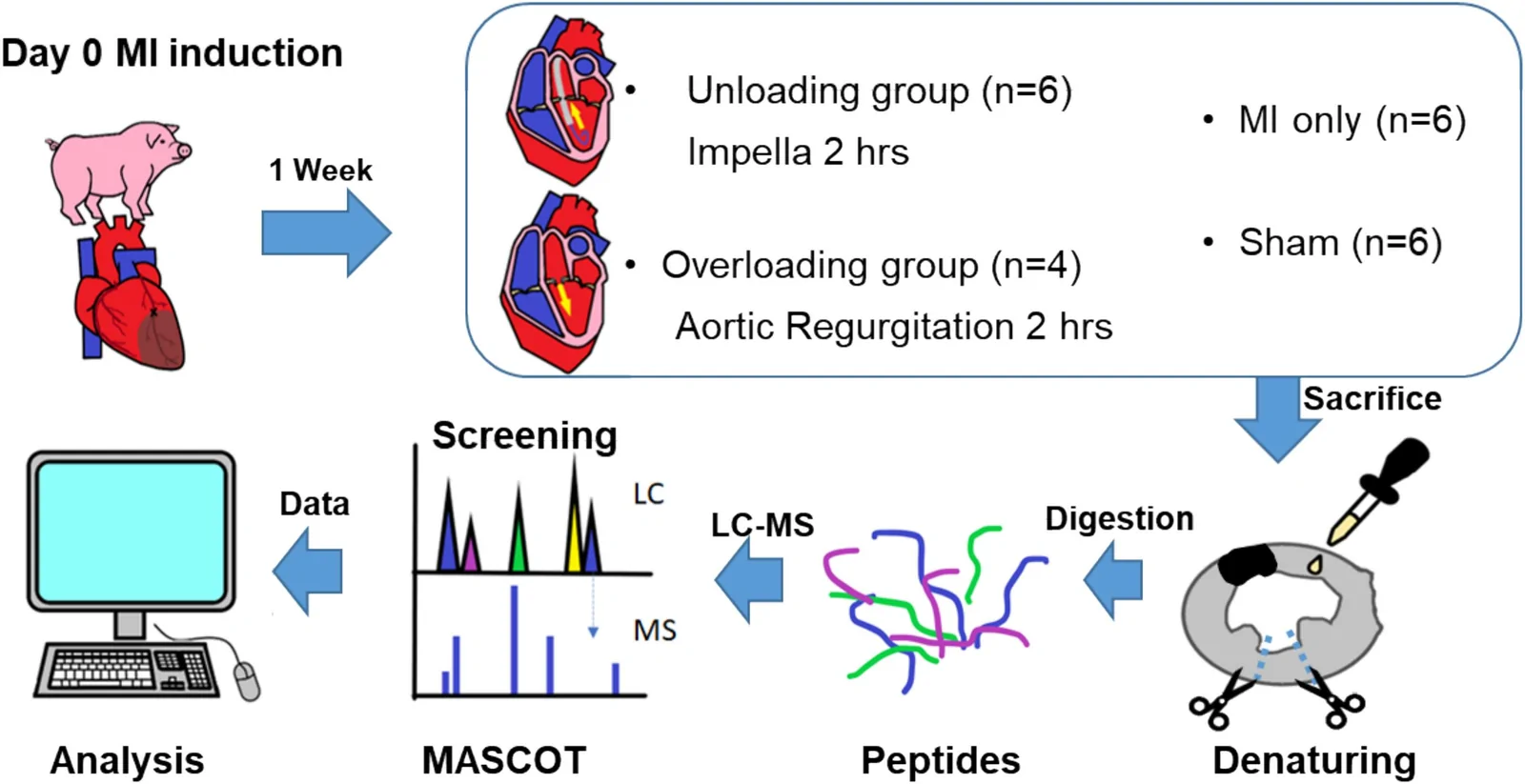
Experimental workflow for the analysis of cardiac proteins in the unloaded and overloaded states. Two samples were collected from the non-ischemic remote regions in each animal and analyzed in duplicate to address reproducibility. MI = myocardial infarction, LC = liquid chromatography, MS = mass spectrometry.

### 2.2 Proteomics

Two pieces (2 technical replicates) of fresh non-ischemic remote myocardial tissues (1 cm^3^) were taken from the LV of each animal and analyzed using previously described proteomic methods [15], [16], [17]. In brief, ECM proteins were extracted from the samples by serial incubations using 0.5 M NaCl (0.5 M NaCl, 10 mM Tris-HCl, 25 mM EDTA, supplemented with protease inhibitors and phosphatase inhibitors, pH = 7.5), 0.1% SDS (0.1% SDS, 25 mM EDTA, supplemented with protease and phosphatase inhibitors), and 4 M guanidine-HCl (4 M Guanidine-HCl, 50 mM sodium acetate, 25 mM EDTA, with protease and phosphatase inhibitors, pH = 5.8) to decellularize and sequentially extract ECM proteins. SDS fractions were used for proteomic experiment. Protein concentration was measured using BCA protein assay (Thermo) and 20 μg of protein from each sample were denatured using 6 M urea, 2 M thiourea, reduced by 10 mM DTT for 1 h at 37C, and alkylated by 50 mM iodoacetamide for 1 h at RT in dark. After acetone precipitation overnight, the protein pellets were resuspended in 0.1 M Triethylammonium bicarbonate (TEAB, pH = 8.5) and digested by trypsin (Thermo) at the ratio of 1:50 overnight. After C18 cleanup using Bravo AssayMAP robot (Agilent), the peptides were separated by nanoflow high performance liquid chromatography (HPLC) (U3000 RSLCnano, Thermo) and identified by mass spectrometry using a Q Exactive HF Orbitrap mass spectrometer (Thermo) with Top15 method. The samples were injected and loaded into a trap cartridge (PepMap, C18, 300um x 5 mm, Thermo) using mobile phase A (0.1% formic acid) at 25 μL/min for 3 min. Then the trap cartridge was connected to an analytical column (PepMap, C18, EasySpray 75 μm x 500 mm, Thermo) and the peptides were eluted at 250 nL/min using the following gradient: 0-5 min, 4%–10% B; 5-75 min, 10%–30% B; 75-80 min, 30%–40% B; 80-85 min, 40%–99% B; 85-90 min, 99% B; 90 − 120 min, 4% B; mobile phase B is 80% acetonitrile with 0.1% formic acid. The full MS data was acquired using an Orbitrap with scan range *m*/*z* 350–1600 and resolution 60,000. The MS2 spectra were collected on the 15 most abundant precursor peaks from the full MS scan after high-energy collisional dissociation (HCD) with collision energy 28, using the orbitrap and resolution 15,000. The dynamic exclusion and lock mass using *m*/*z* 445.1200 were applied. The raw files were searched against a UniProt pig 1 gene 1 protein database (version 2026_02, 22803 protein entries) using Proteome Discoverer (version 3.2) and SequestHT algorithm. The following searching parameters were used: trypsin with 2 missed cleavages was allowed, precursor mass tolerance = 10 ppm, fragment mass tolerance = 20mmu, carbamidomethylation on cysteine as static modification, oxidation on methionine as dynamic modification. Precursor peak intensity was used as quantitation value Protein abundances were calculated based on all quantified peptides from the protein and normalized to the total protein abundance of each sample. Proteins with high confidence (FDR < 0.01) were exported for further statistical analysis.

### 2.3 Differential protein expression and statistical analysis

Protein abundance data were log₂-transformed prior to statistical analysis. To identify proteins differentially expressed across experimental conditions, all quantified proteins were analyzed using a four-group linear modeling framework implemented in the LIMMA package with empirical Bayes variance moderation. The experimental groups included Sham, MI only, Overloading, and Unloading.

Overall group differences were assessed using a moderated analysis of variance (ANOVA), and resulting *p*-values were adjusted for multiple testing using the Benjamini–Hochberg false discovery rate (BH FDR) procedure. Proteins with BH FDR < 0.05 were considered statistically significant.

To further characterize proteomic differences between specific experimental conditions, pairwise differential expression analyses were performed using LIMMA moderated *t*-tests for all biologically relevant comparisons (Sham vs MI, Sham vs Overloading, Sham vs Unloading, MI vs Overloading, MI vs Unloading, and Overloading vs Unloading). Resulting *P* values were adjusted for multiple testing using the BH FDR procedure, with BH FDR < 0.05 considered statistically significant. Volcano plots were generated using SRplot, a web-based platform for data visualization and graphing [18], and displayed log₂ fold change versus −log₁₀(BH-adjusted *P* value). Vertical reference lines were set at log₂ fold changes of ±0.585 (corresponding to a 1.5-fold change), and the horizontal reference line indicates BH FDR = 0.05. Hierarchical clustering was performed using the top 50 proteins ranked by four-group moderated ANOVA for descriptive visualization of overall proteomic patterns. Protein abundance values were transformed to row-wise Z-scores prior to clustering, and both proteins and samples were clustered using Euclidean distance and complete linkage. Heatmaps were generated to visualize protein expression patterns across the four experimental groups. Inclusion of the top 50 ANOVA-ranked proteins was not considered evidence of statistical significance unless the BH FDR < 0.05 criterion was met.

### 2.4 Functional enrichment analysis and association network analyses

Functional enrichment analysis was performed using g:Profiler with the *Sus scrofa* reference database using the seven proteins that met the four-group ANOVA significance threshold (BH FDR < 0.05). The statistical domain scope was set to all known genes, and enrichment *P* values were adjusted using the BH FDR procedure. Terms with BH FDR < 0.05 were considered significantly enriched. Representative non-redundant terms from Gene Ontology (GO), Reactome, and KEGG were selected for visualization.

Functional association networks were generated using STRING (version 12.0) with the *Sus scrofa* reference database. The seven ANOVA-significant proteins were submitted to STRING, of which six were successfully mapped; A0A8W4FCZ3 was not mapped. The full STRING network was generated using a minimum required interaction score of 0.40 (medium confidence), with no additional interactors added. Network edges were displayed according to supporting evidence.

### 2.5 Exploratory identification of load-associated candidate proteins

In addition to proteins meeting the conventional four-group ANOVA significance threshold (BH FDR < 0.05), an exploratory screening was performed to identify potential LV load-associated candidate proteins. Proteins with ANOVA FDR < 0.25 and nominal *P* < 0.05 for the Overloading versus Unloading comparison were considered exploratory load-associated candidates. The nominal *P* values for the Overloading versus Unloading comparison were derived from the LIMMA moderated *t*-test described above. This relaxed threshold was used solely for hypothesis-generating candidate prioritization and not for statistical inference.

For each protein, the disease effect was defined as the log_2_ fold change for the MI vs Sham comparison, whereas the LV load effect was defined as the log_2_ fold change for the Overloading vs Unloading comparison. The seven ANOVA-significant proteins and exploratory LV load-associated candidates were visualized using a heatmap and a two-dimensional comparison of disease and LV load effects.

### 2.6 Human DCM transcriptomic analysis

To examine the disease relevance of representative exploratory candidates in human myocardium, transcript expression of ITIH1 and MYOZ2 was evaluated using the publicly available human dilated cardiomyopathy (DCM) RNA-seq dataset GSE141910. Sample-level expression values were obtained for non-failing (NF; *n* = 161) and DCM (*n* = 161) hearts. Differences in transcript expression between the non-failing and DCM groups were assessed using two-tailed Welch's *t*-test.

## 3 Results

### 3.1 Global proteomic profiling reveals differential protein expression across MI, overloading, and unloading

Quantitative proteomic analysis was performed using non-ischemic remote LV myocardium obtained from the Sham, MI, Overloading, and Unloading groups. Average scar size and cardiac parameters before and during unloading/overloading in the studied animals are shown in Table 1. A total of 1713 proteins were identified, of which 1552 proteins were retained for statistical analysis across all samples. The complete results of the four-group moderated ANOVA and pairwise differential expression analyses for all quantified proteins are provided in Supplementary Table S1. Four-group LIMMA moderated ANOVA identified 7 proteins that remained significant after BH FDR correction (adjusted *P* < 0.05) (Table 2). The overall analytical workflow is summarized in Fig. 2.

Pairwise differential expression analyses were subsequently performed using LIMMA moderated *t*-tests to compare protein abundance between experimental groups. Volcano plots summarized protein expression changes across the six pairwise comparisons (Fig. 3a). To further visualize protein expression patterns across all four experimental groups, the top 50 proteins ranked by four-group moderated ANOVA were subjected to hierarchical clustering. The resulting heatmap identified distinct expression patterns across the Sham, MI, Overloading, and Unloading groups (Fig. 3b). This analysis was used for descriptive visualization of overall proteomic patterns rather than for identification of statistically significant proteins. Accordingly, inclusion among the top 50 ANOVA-ranked proteins was not considered evidence of statistical significance unless the BH FDR < 0.05 criterion was met.

**Fig. 2:**
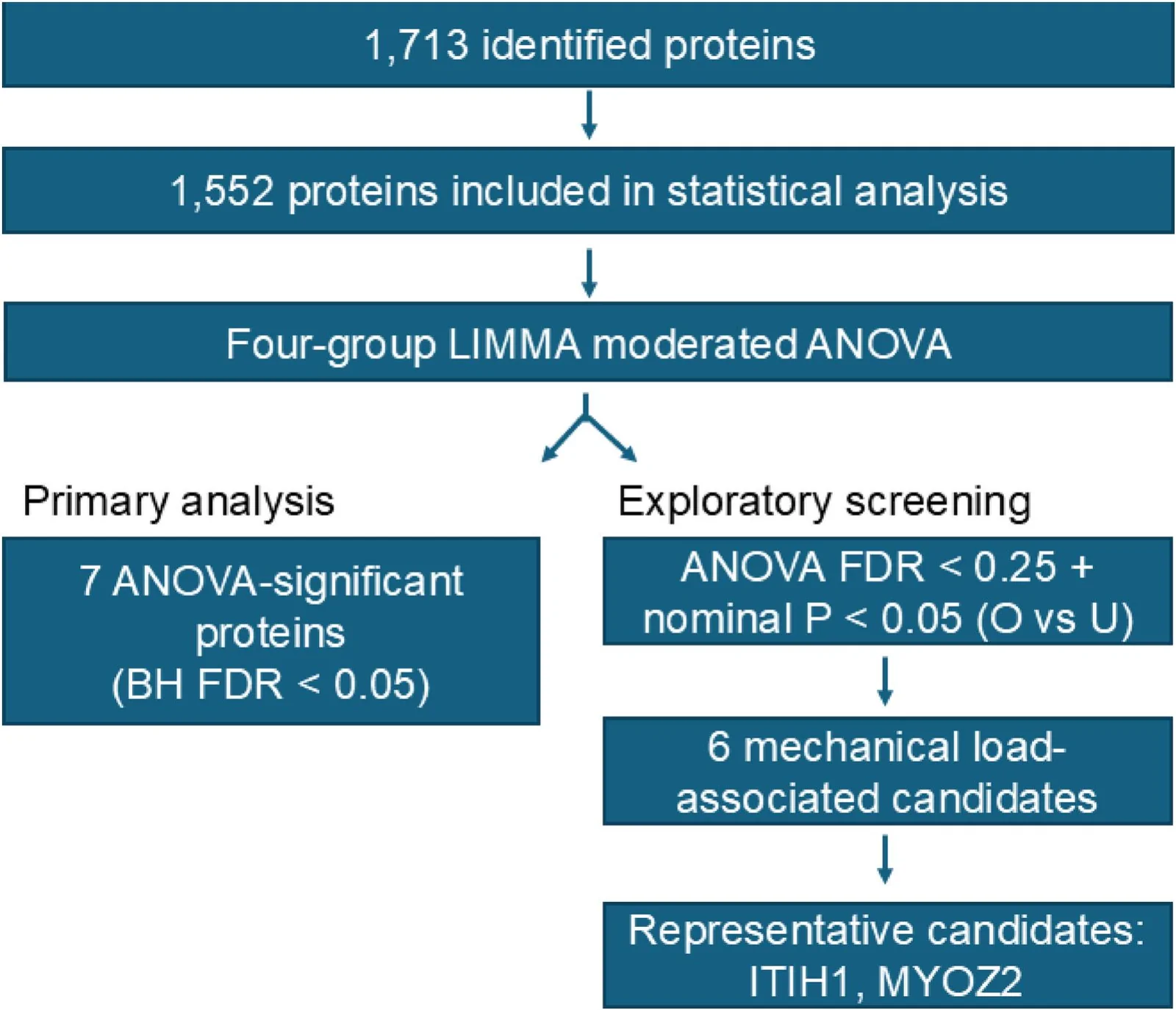
Proteomic analysis workflow and candidate selection strategy. A total of 1713 proteins were identified, of which 1552 were included in the statistical analysis. A four-group LIMMA moderated ANOVA was performed as the primary analysis, identifying seven proteins at BH FDR < 0.05. In parallel, an exploratory screening was performed to identify potential mechanical load-associated candidates using ANOVA FDR < 0.25 together with nominal *P* < 0.05 for the Overloading vs Unloading comparison. Six proteins met these exploratory criteria, from which ITIH1 and MYOZ2 were selected as representative candidates for further visualization. O = Overloading, U = Unloading, BH FDR = Benjamini–Hochberg false discovery rate, MI = myocardial infarction.

**Table 1:** Scar size and cardiac parameters before and during LV unloading/overloading.

	Unloading	Overloading	MI-only	p-Value (ANOVA)
Scar size (% of LV)	21.7 ± 4.2	23.8 ± 2.4	26.4 ± 7.3	0.35
Post MI, pre unloading/overloading				
3D end-diastolic volume (mL)	132 ± 29	105 ± 7	96 ± 19	0.03
3D end-systolic volume (mL)	81 ± 21	54 ± 10	57 ± 18	0.05
3D ejection fraction (%)	39 ± 5	48 ± 10	41 ± 12	0.37
End-diastolic pressure (mmHg)	19.5 ± 4.5	17.0 ± 8.1	18.1 ± 10.3	0.88
dP/dt maximum (mmHg/mL)	2081 ± 674	2049 ± 908	1689 ± 442	0.61
dP/dt minimum (mmHg/mL)	−1947 ± 499	−1894 ± 581	−1688 ± 374	0.67
During unloading/overloading				
End-diastolic pressure (mmHg)	12.1 ± 4.3	23.9 ± 7.8		0.01
dP/dt maximum (mmHg/mL)	1293 ± 320	1727 ± 276		0.06
dP/dt minimum (mmHg/mL)	−1360 ± 428	−1263 ± 121		0.68

LV = left ventricular, MI = myocardial infarction.

**Table 2:** Proteins significantly altered by four-group LIMMA moderated ANOVA (BH FDR < 0.05).

Accession	Gene	AveExpr	F	P value	BH FDR	Description
A0A8W4FCZ3	–	26.41293659	12.667253	4.5502E−05	0.0374292	Laminin G domain-containing protein
A0A5G2R7M3	ITIH4	23.2421341	12.585476	4.82335E−05	0.0374292	Inter-alpha-trypsin inhibitor heavy chain 4
A0A480KXA1	MYOM1	28.13293403	10.993425	0.000118165	0.04502152	Myomesin-1
A0A287AT48	LOC100153899	23.80435592	10.755779	0.000136213	0.04502152	Serpin domain-containing protein
A0A287BMX9	NID1	27.79316943	10.651696	0.000145044	0.04502152	Nidogen 1
F1SCC7	LOC396684	22.81651112	10.280339	0.000181992	0.04541696	Serpin domain-containing protein
A0A287A718	CP	21.86834215	10.089643	0.000204845	0.04541696	Ceruloplasmin

Proteins showing significant differences among the Sham, myocardial infarction (MI), Overloading, and Unloading groups were identified using four-group LIMMA moderated ANOVA with Benjamini–Hochberg false discovery rate (BH FDR) correction. Only proteins with adjusted *P* values <0.05 are shown. Proteins are ranked according to the moderated F statistic. AveExpr represents the average log₂-normalized protein abundance across all samples. For proteins without assigned gene symbols, the corresponding accession identifiers are shown.

**Fig. 3:**
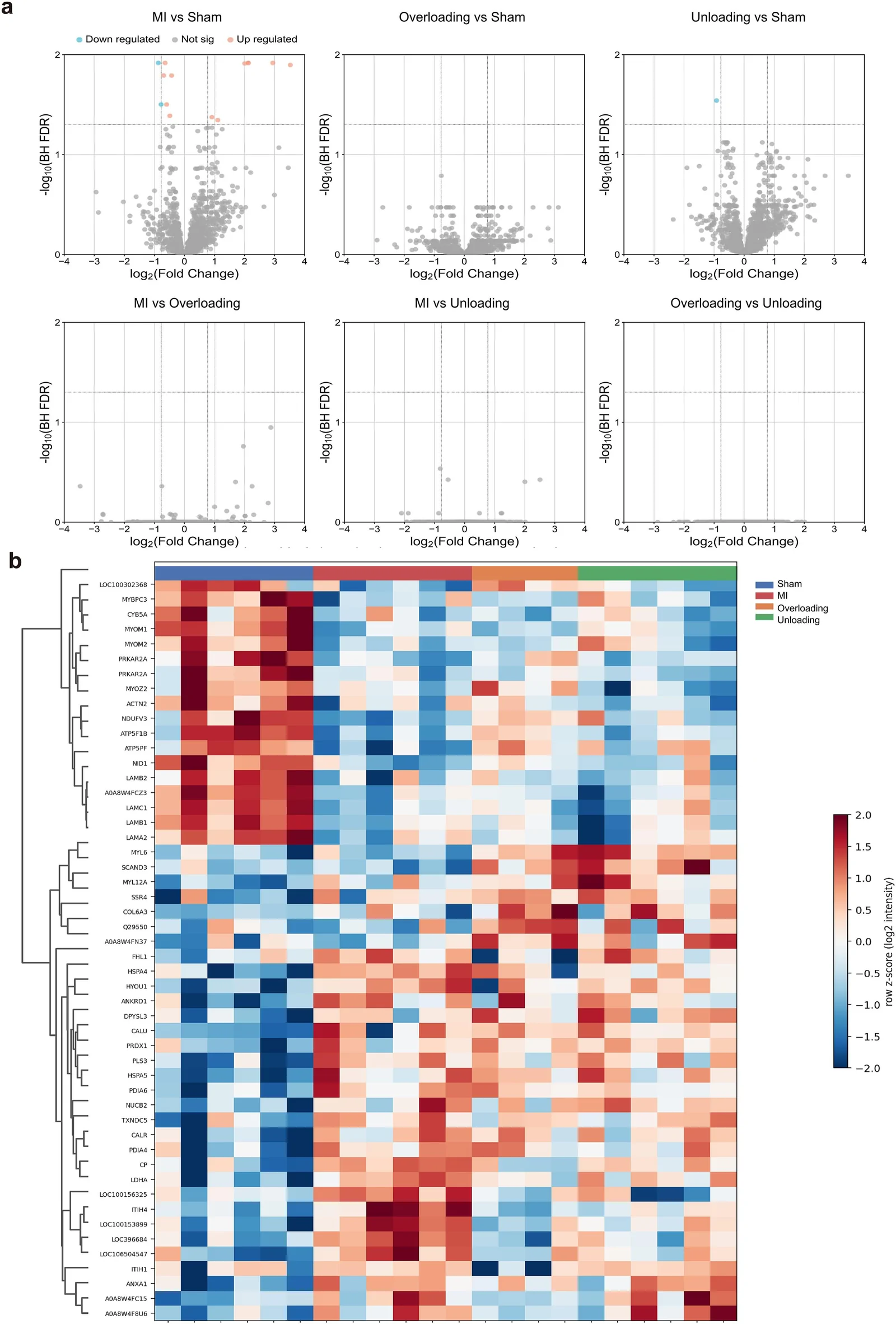
Pairwise proteomic comparisons and hierarchical clustering of ANOVA-ranked proteins. (a) Volcano plots showing pairwise proteomic comparisons between Sham, MI, Overloading, and Unloading groups. The x-axis represents log_2_ fold change and the y-axis represents −log_10_(BH-adjusted *P* value). Vertical dashed lines indicate log_2_ fold-change values of ±0.585, corresponding to a 1.5-fold change, and the horizontal dashed line indicates a BH FDR of 0.05. Proteins meeting BH FDR < 0.05 are highlighted according to the direction of change; all other proteins are shown in gray. (b) Hierarchical clustering heatmap of the top 50 proteins ranked by the four-group LIMMA moderated ANOVA. Protein abundance is displayed as row-wise z-scores of log_2_-transformed intensity. The heatmap is shown for descriptive visualization of the overall proteomic patterns across the four experimental groups; inclusion among the top 50 does not indicate statistical significance after multiple-testing correction.

### 3.2 Functional characteristics of ANOVA-significant proteins

The four-group LIMMA moderated ANOVA identified seven proteins that remained significant after multiple-testing correction (BH FDR < 0.05): A0A8W4FCZ3, ITIH4, MYOM1, LOC100153899, NID1, LOC396684, and CP (Table 2). These proteins included a laminin G domain-containing protein (A0A8W4FCZ3), inter-alpha-trypsin inhibitor heavy chain 4 (ITIH4), myomesin-1 (MYOM1), nidogen-1 (NID1), two serpin domain-containing proteins (LOC100153899 and LOC396684), and ceruloplasmin (CP). In the MI versus Sham comparison, ITIH4 (log₂FC = 2.94), LOC100153899 (log₂FC = 2.13), CP (log₂FC = 2.12), and LOC396684 (log₂FC = 2.01) were increased, whereas A0A8W4FCZ3 (log₂FC = −0.87), MYOM1 (log₂FC = −0.69), and NID1 (log₂FC = −0.64) were decreased. All seven proteins remained significant after BH FDR correction in this pairwise comparison. A0A8W4FCZ3 was also significantly decreased in the Unloading versus Sham comparison (log₂FC = −0.920; BH FDR = 0.0288), whereas none of the seven proteins reached BH FDR < 0.05 in the Overloading versus Unloading comparison. Thus, the ANOVA-significant protein set showed heterogeneous expression changes following MI, whereas direct differences between Overloading and Unloading were not statistically significant after multiple-testing correction.

Functional enrichment of the seven ANOVA-significant proteins revealed associations primarily with extracellular matrix and protease-regulatory functions, together with sarcomeric and iron-related processes (Supplementary Fig. S1A). Among the enriched molecular function terms, serine-type endopeptidase inhibitor activity showed significant enrichment (BH FDR = 3.67 × 10^−5^), together with related terms involving peptidase and enzyme inhibitor activity. Additional molecular function terms included ferroxidase activity and laminin−1 binding (BH FDR = 3.60 × 10^−3^ for each), as well as proteoglycan binding, collagen binding, and extracellular matrix structural constituent. Cellular component analysis identified significant enrichment of extracellular region and chromaffin granule terms (BH FDR = 8.90 × 10^−5^ for each), as well as M-band localization (BH FDR = 1.51 × 10^−2^). Reactome analysis further identified laminin interactions (BH FDR = 3.58 × 10^−5^) and extracellular matrix organization (BH FDR = 3.46 × 10^−3^). KEGG pathway analysis additionally identified ferroptosis among the significantly enriched pathways (BH FDR = 1.12 × 10^−2^). Representative non-redundant enrichment terms are shown in Supplementary Fig. S1A.

Functional associations among the seven ANOVA-significant proteins were further examined using STRING analysis (Supplementary Fig. S1B). Six of the seven proteins were mapped to the *Sus scrofa* STRING network, whereas A0A8W4FCZ3 was not mapped. The resulting network contained six nodes and three edges, compared with zero edges expected by chance (PPI enrichment *P* = 2.85 × 10^−6^). The observed interactions involved ITIH4, LOC100153899, and CP, whereas MYOM1, NID1, and LOC396684 remained disconnected at the applied interaction threshold. Thus, the ANOVA-significant proteins did not form a broadly interconnected network, although a subset comprising ITIH4, LOC100153899, and CP showed functional connectivity.

### 3.3 Exploratory identification of LV load-associated candidate proteins

In parallel with the primary analysis, exploratory screening using ANOVA FDR < 0.25 and nominal *P* < 0.05 for the Overloading versus Unloading comparison identified six LV load-associated candidate proteins (Table 3). These candidates included ITIH1, MYOZ2, FHL1, A0A8W4FC15, LOC100302368, and A0A8W4F8U6, with ANOVA FDR values ranging from 0.141 to 0.226. The nominal *P* values for the Overloading versus Unloading comparison ranged from 0.0022 to 0.0324. Among these candidates, ITIH1 showed the largest LV load contrast (|log₂FC| = 2.88), followed by FHL1 (|log₂FC| = 2.00), whereas the remaining candidates showed smaller differences between the Overloading and Unloading groups. The seven ANOVA-significant proteins and six exploratory LV load-associated candidates were subsequently visualized together to compare their expression patterns across the four experimental groups. Heatmap visualization showed heterogeneous abundance patterns among the 13 proteins across Sham, MI, Overloading, and Unloading conditions (Fig. 4a).

**Table 3:** Primary ANOVA-significant proteins and exploratory mechanical load-associated candidate proteins.

Category	Gene	Accession	Description	Putative biological role	ANOVA FDR	MI effect (log2FC, MI vs Sham)	Mechanical load contrast (log2FC, overloading vs unloading)	Nominal *P* value (overloading vs unloading)	Representative candidate
ANOVA-significant	ITIH4	A0A5G2R7M3	Inter-alpha-trypsin inhibitor heavy chain 4	Acute-phase response; extracellular matrix stabilization	0.0374292	2.940107529	0.87243962	0.15603432	
ANOVA-significant	LOC100153899	A0A287AT48	Serpin domain-containing protein	Serine protease inhibition; inflammatory response	0.04502152	2.128539087	0.56998783	0.206056406	
ANOVA-significant	CP	A0A287A718	Ceruloplasmin	Iron homeostasis; antioxidant defense	0.04541696	2.119063359	0.74657278	0.112650992	
ANOVA-significant	LOC396684	F1SCC7	Serpin domain-containing protein	Serine protease inhibition; extracellular homeostasis	0.04541696	2.007020901	0.72477627	0.11937005	
ANOVA-significant	A0A8W4FCZ3	A0A8W4FCZ3	Laminin G domain-containing protein	Extracellular matrix organization	0.0374292	0.868345263	0.14043497	0.469065728	
ANOVA-significant	MYOM1	A0A480KXA1	Myomesin-1	Sarcomere structural organization	0.04502152	0.689875826	0.130816	0.430357374	
ANOVA-significant	NID1	A0A287BMX9	Nidogen 1	Basement membrane organization	0.04502152	0.643883796	0.19062209	0.187951734	
Mechanical load-associated (exploratory)	ITIH1	Q29052	Inter-alpha-trypsin inhibitor heavy chain H1	Extracellular matrix stabilization; tissue remodeling	0.22620245	1.461670611	2.878921	0.002215831	Yes (Fig. 4)
Mechanical load-associated (exploratory)	MYOZ2	A0A8W4FPV8	Myozenin 2	Z-disc organization; mechanotransduction	0.1408651	0.354572918	0.46635162	0.003720358	Yes (Fig. 4)
Mechanical load-associated (exploratory)	FHL1	A0A4X1SFB6	Four and a half LIM domains protein 1	LIM-domain scaffold protein; muscle structural remodeling	0.21894679	1.347851807	2.00171364	0.004368556	
Mechanical load-associated (exploratory)	A0A8W4FC15	A0A8W4FC15	Ig-like domain-containing protein	Cell adhesion; extracellular interaction (putative)	0.15869928	1.090215058	0.97683667	0.014019659	
Mechanical load-associated (exploratory)	LOC100302368	A0A287B5W2	trypsin	Proteolysis; extracellular protein turnover	0.15791711	0.48503286	0.33975763	0.027167138	
Mechanical load-associated (exploratory)	A0A8W4F8U6	A0A8W4F8U6	Ig-like domain-containing protein	Cell adhesion; extracellular interaction (putative)	0.21916979	0.762129277	0.61745559	0.032366421	

ANOVA-significant proteins met the conventional four-group ANOVA threshold of BH FDR < 0.05. Mechanical load-associated proteins were identified using an exploratory screening criterion of ANOVA FDR < 0.25 and nominal *P* < 0.05 for the overloading versus unloading comparison and should be considered hypothesis-generating candidates.

**Fig. 4:**
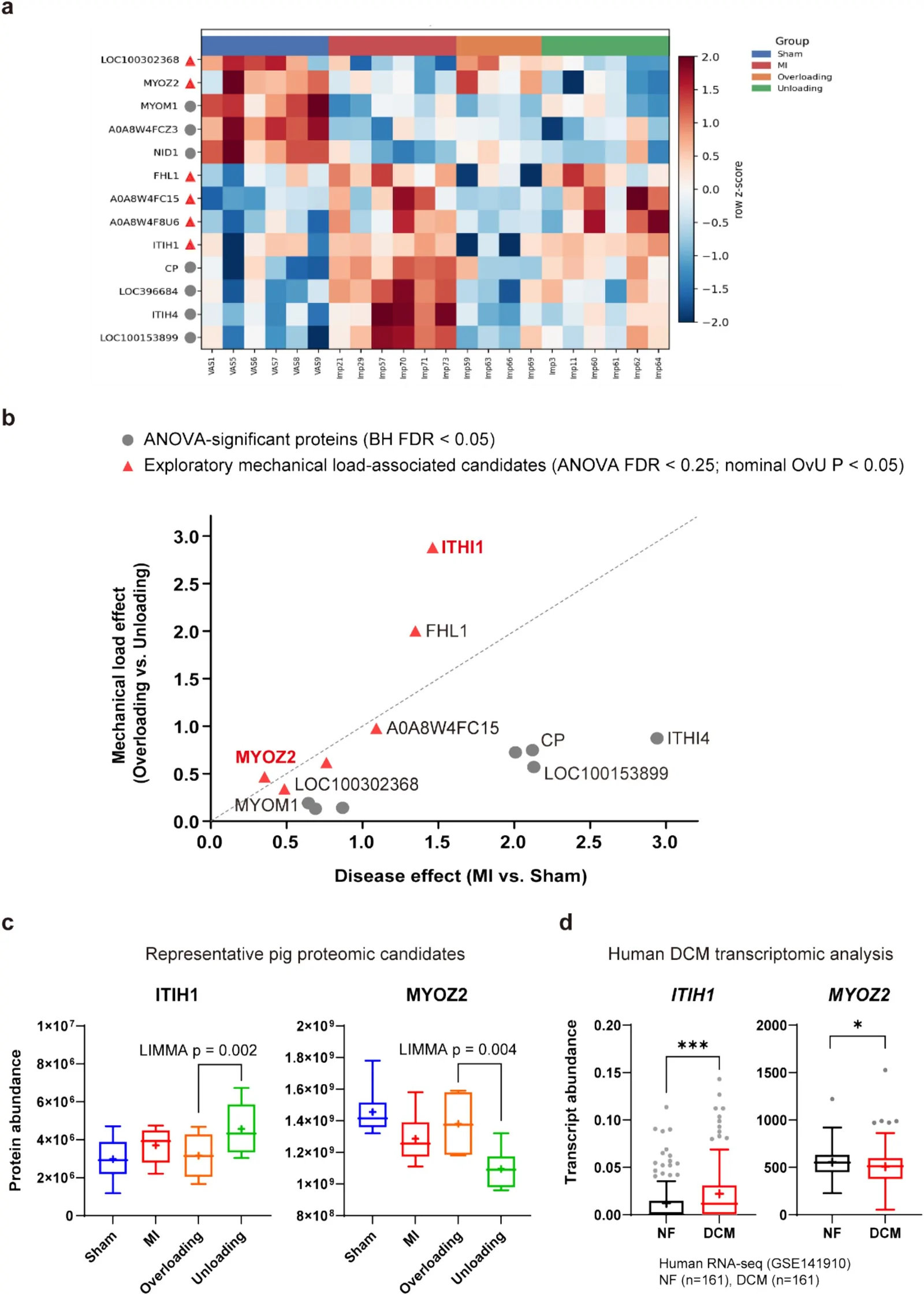
Primary ANOVA-significant proteins and exploratory mechanical load-associated candidates. (a) Heatmap showing the abundance patterns of seven ANOVA-significant proteins (BH FDR < 0.05; gray circles) and six exploratory mechanical load-associated candidates (red triangles) across the Sham, MI, Overloading, and Unloading groups. Protein abundance is displayed as row-wise z-scores. (b) Relationship between the disease effect (MI vs Sham) and mechanical load effect (Overloading vs Unloading) for the proteins shown in (a). Gray circles indicate ANOVA-significant proteins (BH FDR < 0.05), whereas red triangles indicate exploratory mechanical load-associated candidates identified using ANOVA FDR < 0.25 and nominal *P* < 0.05 for the Overloading versus Unloading comparison. The diagonal dashed line indicates equal magnitudes of disease and mechanical load effects. ITIH1 and MYOZ2 are highlighted as representative exploratory candidates. (c) Protein abundance of ITIH1 and MYOZ2 across the four experimental groups. Pairwise comparisons between Overloading and Unloading were performed using LIMMA moderated *t*-tests; nominal *P* values are shown. (d) Transcript abundance of ITIH1 and MYOZ2 in non-failing (NF) and dilated cardiomyopathy (DCM) human hearts from the GSE141910 dataset. *P* values are indicated by asterisks as shown. (For interpretation of the references to color in this figure legend, the reader is referred to the web version of this article.)

To further examine the relationship between disease-associated and LV load-associated changes, the MI versus Sham contrast was plotted against the Overloading versus Unloading contrast for the seven ANOVA-significant proteins and six exploratory candidates (Fig. 4b). This visualization showed that the magnitude of the two effects varied among individual proteins. Among the exploratory candidates, ITIH1 showed relatively large changes along both axes, with higher abundance in MI than Sham (log₂FC = 1.46) and in Overloading than Unloading (log₂FC = 2.88). In contrast, MYOZ2 showed smaller changes, with slightly higher abundance in MI than Sham (log₂FC = 0.35) and in Overloading than Unloading (log₂FC = 0.47). Based on these distinct patterns of disease- and LV load-associated changes, ITIH1 and MYOZ2 were selected as representative exploratory candidates for further characterization. Protein abundance across the four experimental groups was subsequently examined for ITIH1 and MYOZ2 (Fig. 4c). Consistent with the exploratory screening analysis, the Overloading versus Unloading comparison showed nominal significance for both MYOZ2 (*P* = 0.0037) and ITIH1 (*P* = 0.0022) based on the LIMMA moderated *t*-test.

To provide additional human disease-related context for these exploratory candidates, ITIH1 and MYOZ2 transcript expression was evaluated in an independent human DCM RNA-seq dataset (GSE141910). ITIH1 transcript levels were significantly increased in DCM compared with non-failing hearts (*P* = 0.0004), whereas MYOZ2 transcript levels were significantly reduced (*P* = 0.0119) (Fig. 4d). Thus, the two representative candidates showed opposite disease-associated transcript expression patterns in the independent human DCM dataset.

## 4 Discussion

Using unique pig models of acutely altered cardiac load conditions, duplicate samples from each animal to increase rigor, and a robust proteomic analysis, we characterized protein remodeling of non-ischemic remote myocardium in ischemic heart failure. While several studies investigated protein changes in patients supported by long-term LVAD [2], [3], [4], [12], there has been no report that examined the impact of acute mechanical unloading. Results of our study indicate that protein responses to 2 h of acute load alteration is much less significant compared to the impact of MI. Nevertheless, exploratory analysis identified LV load-associated candidates, including ITIH1 and MYOZ2, providing a basis for further investigation of early molecular responses to altered cardiac load. Given the increased use of catheter-based LV assist devices in clinics to treat cardiogenic shock and heart failure [19], our study provides new insights in therapeutic exploration to enhance myocardial protection and recovery using these devices.

The MI-associated proteomic changes were consistent with remodeling across multiple myocardial compartments. Changes in proteins related to sarcomeric organization, mitochondrial metabolism, glycolysis, and extracellular matrix components were observed in the MI vs Sham comparison. Previous proteomic studies in porcine myocardial ischemia/reperfusion have similarly demonstrated extensive remodeling of the cardiac extracellular matrix [15]. Notably, the decrease in MYOM1 is consistent with previous proteomic observations in porcine remote myocardium following myocardial ischemia/reperfusion, where MYOM1 showed a transient decrease during early reperfusion [20]. The seven proteins that remained significant after multiple-testing correction further highlighted extracellular matrix and protease-regulatory processes, together with sarcomeric and iron-related functions. Together, these findings suggest that early remodeling of non-ischemic myocardium involves changes across extracellular, contractile, and metabolic compartments.

In our exploratory analysis, we found ITIH1 and MYOZ2 as candidate proteins that might respond to altered LV load. MYOZ2 (calsarcin-1) is a Z-disc-associated protein linked to calcineurin signaling and sarcomeric stress responses, providing a potential connection between LV load and cardiomyocyte signaling [21], [22]. Meanwhile, ITIH1 belongs to the inter-α-trypsin inhibitor family and has been implicated in extracellular matrix stabilization and tissue remodeling, suggesting a potentially different extracellular component of the response [22]. Independent human transcriptomic data in human DCM study further supported the translational relevance of ITIH1 and MYOZ2 in the context of human cardiac disease. Thus, while differences in species, heart failure phenotype and exploratory nature of our study warrant careful interpretation, ITIH1 and MYOZ2 may represent interesting candidates for further investigation of extracellular and sarcomeric responses to altered myocardial load.

Several limitations should be acknowledged. First, the proteomic analysis was performed in a porcine model with a limited number of biological replicates. No proteins reached BH FDR < 0.05 in the direct Overloading vs Unloading comparison, while the six LV load-associated candidates were identified using relaxed exploratory criteria (ANOVA FDR < 0.25 and nominal *P* < 0.05 for Overloading versus Unloading). These candidates should therefore be considered hypothesis-generating. However, the LV tissues from clinically relevant large-animal model combined with duplicate sample processing were used to strengthen the rigor and our data provides unique results within the constraints of experimental setting. Second, although independent human transcriptomic analysis supported the translational relevance of MYOZ2 and provided supportive evidence for ITIH1, validation at the protein level in human myocardial tissue was not performed. Third, the unloading/overloading period was short compared to the clinical application for cardiogenic shock. Cardiac protein changes after longer duration of unloading might provide additional insights, but this was not available due to the logistical limitations associated with large animal experiments in the current design. Our cardiac proteomic results are therefore a snapshot of an early timepoint after the load alterations and future studies are needed to examine the impact of unloading duration on myocardial proteomic remodeling.

## 5 Conclusions

We characterized proteomic remodeling of the non-ischemic remote myocardium in response to MI and altered acute LV load. Our unique analysis framework identified exploratory load-associated candidate proteins that can provide insights into further exploration.

## CRediT authorship contribution statement

**Spyros A. Mavropoulos:** Writing – review & editing, Writing – original draft, Visualization, Validation, Methodology, Investigation, Formal analysis, Data curation, Conceptualization. **Changwon Kho:** Writing – review & editing, Writing – original draft, Visualization, Software, Investigation, Funding acquisition, Formal analysis. **Marika Fava:** Writing – review & editing, Resources, Methodology, Investigation, Data curation, Conceptualization. **Ahyoung Lee:** Writing – review & editing, Visualization, Software, Methodology, Investigation, Formal analysis. **Kyra Leonard:** Writing – review & editing, Methodology, Investigation, Data curation. **Samta Veera:** Writing – original draft, Validation, Methodology, Investigation, Data curation. **Tomoki Sakata:** Writing – review & editing, Methodology, Investigation, Data curation. **Renata Mazurek:** Writing – review & editing, Methodology, Investigation, Data curation. **Anjali J. Ravichandran:** Writing – review & editing, Investigation, Data curation. **Jonas M. Marx:** Writing – review & editing, Investigation, Data curation. **Xiaoke Yin:** Writing – review & editing, Visualization, Software, Methodology, Investigation, Formal analysis, Data curation. **Manuel Mayr:** Writing – review & editing, Supervision, Methodology, Investigation, Funding acquisition, Formal analysis, Data curation, Conceptualization. **Kiyotake Ishikawa:** Writing – review & editing, Writing – original draft, Visualization, Validation, Supervision, Software, Resources, Project administration, Methodology, Investigation, Funding acquisition, Formal analysis, Data curation, Conceptualization.

## Sources of funding

This work was supported by 10.13039/100000002NIH R01 HL139963 (K.I.), HL173593 (K.I.) and 10.13039/501100003725National Research Foundation of Korea grant funded by the Korean government: Grant No. RS-2024-00339639 (C.K.). F S.A.M. was supported by 10.13039/100000002National Institutes of Health T32HL007824-23. We would like to acknowledge the equipment funding from the NIH on the animal cathlab C-arm (S10OD034330).

## Declaration of competing interest

The authors declare the following financial interests/personal relationships which may be considered as potential competing interests: Kiyotake Ishikawa reports financial support was provided by National Heart Lung and Blood Institute. Spyros Mavropoulos reports financial support was provided by National Heart Lung and Blood Institute. Changwon Kho reports financial support was provided by National Research Foundation of Korea. Kiyotake Ishikawa reports a relationship with AbioMed Inc. that includes: consulting or advisory, funding grants, non-financial support, and speaking and lecture fees. Tomoki Sakata reports a relationship with AbioMed Inc. that includes: funding grants. KIYOTAKE ISHIKAWA has patent # 20200305888 issued to Kiyotake Ishikawa. None If there are other authors, they declare that they have no known competing financial interests or personal relationships that could have appeared to influence the work reported in this paper.

## Figures and Tables

**Supplementary Fig. S1:**
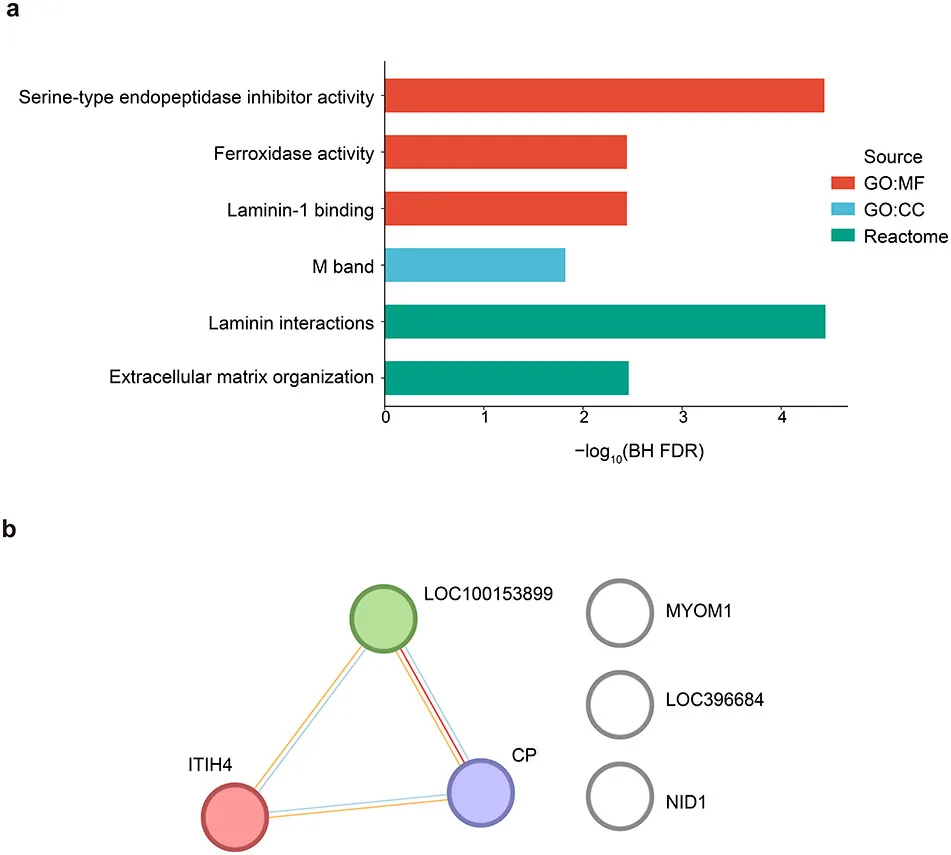
Functional characterization of the seven ANOVA-significant proteins (a) Functional enrichment analysis of the seven proteins meeting the four-group LIMMA moderated ANOVA significance threshold (BH FDR < 0.05) using g:Profiler with *Sus scrofa* annotations. The statistical domain scope was set to all known genes, and significance was determined using Benjamini–Hochberg FDR correction. Representative non-redundant significantly enriched terms (BH FDR < 0.05) are shown. (b) STRING functional association network of the ANOVA-significant proteins using the *Sus scrofa* database with a minimum interaction score of 0.40 (medium confidence). Six of the seven proteins were mapped to the STRING network, which contained six nodes and three edges (PPI enrichment *P* = 2.85 × 10^−6^). A0A8W4FCZ3 was not mapped. LOC100153899 was mapped to SERPINA3-2 in STRING and is labeled according to the identifier used in the proteomic dataset.

## Appendix A Supplementary data

Supplementary Table S1 Complete results of four-group LIMMA moderated ANOVA and pairwise differential expression analyses. The table includes the full normalized protein abundance matrix, protein accession, gene symbol, protein description, moderated ANOVA statistics, and results for all six pairwise comparisons.
